# Comparative Analysis of Techniques for Pit and Fissure Sealant Application to Reduce Microleakage

**DOI:** 10.7759/cureus.54984

**Published:** 2024-02-26

**Authors:** Pranav Patil, Neeta S Padmawar, Divyam Girdhar, Sneha Singh, Yash D Shah, Seema Gupta

**Affiliations:** 1 Department of Conservative Dentistry and Endodontics, Bharati Vidyapeeth Dental College and Hospital, Sangli, IND; 2 Department of Pediatrics and Preventive Dentistry, Rural Dental College, Pravara Institute of Medical Sciences, Loni, IND; 3 Department of Dentistry, School of Medicine, Amrita Vishwa Vidyapeetham, Faridabad, IND; 4 Department of Conservative Dentistry and Endodontics, Rungta College of Dental Sciences and Research, Bhilai, IND; 5 Department of Oral Medicine and Radiology, Jawahar Medical Foundation's Annasaheb Chudaman Patil Memorial Medical College and Hospital, Dhule, IND; 6 Department of Orthodontics, Kothiwal Dental College and Research Centre, Moradabad, IND

**Keywords:** dental fissures, in vitro, caries, dentin bonding agents, pit and fissure sealants

## Abstract

Introduction

In modern dentistry, the focus is more on preventing caries than on treating it, which helps preserve the tooth structure. Pit and fissure sealants (PFS) are the most effective methods for providing a mechanical barrier and avoiding the accumulation of dental plaque in deep pits and fissures, thereby preventing occlusal caries. The present study was conducted to compare the efficiency of dentin bonding agents (DBA) with or without fissurotomy in reducing microleakage before PFS placement.

Materials and methods

A total of 48 freshly extracted premolars were randomly divided into four groups as follows: Group 1, the conventional technique of PFS (Clinpro, 3M ESPE sealant); Group 2, fissurotomy performed before PFS placement; Group 3, Scotchbond Universal Adhesive (3M ESPE DBA) applied before PFS placement; and Group 4, fissurotomy along with DBA was used before PFS placement. The teeth were subjected to thermocycling followed by dye penetration using a 1% solution of methylene blue for 24 hours. All teeth were then assessed for microleakage by a qualitative method using a stereomicroscope at 40X and depth of dye penetration by image analysis. The Kruskal-Wallis test followed by Dunn’s test was used for intergroup comparisons of microleakage scores, and ANOVA followed by Tukey’s test was used for intergroup comparisons of the depth of dye penetration. These analyses were conducted using statistical software (SPSS version 22, Chicago, IL, USA).

Results

Statistically significant differences were observed between the groups in terms of the microleakage scores and depth of dye penetration (p<0.05). The group 4 showed a minimum microleakage score (0.50±0.52), and maximum scores were observed in Group 1 (2.16±0.71). Group 2 showed insignificant differences with groups 3 and 4 for depth of dye penetration (p>0.05). Statistically significant differences were observed between groups 1 and 2, groups 1 and 4, and groups 3 and 4 for the microleakage score (p<0.05).

Conclusion

Fissurotomy with or without DBA significantly reduced microleakage before the PFS placement. Prior use of fourth-generation DBA significantly reduced microleakage compared with PFS placement without the use of DBA.

## Introduction

Despite advancements in dental health worldwide, dental caries continue to be the prevailing chronic childhood ailment on a global scale. Dental caries are more likely to occur in teeth with deep fissures and pits. Conversely, the application of fissure sealants has been established as an effective measure to prevent the formation of caries in these areas and their development on occlusal surfaces [1]. The presence of microleakage within sealants is regarded as a vulnerable aspect that ultimately results in failure owing to the inability to isolate pits and fissures, thus promoting the accumulation of bacteria, nutrients, and acidic metabolic byproducts [2]. However, the effectiveness of pit and fissure sealants (PFSs) may be compromised by technical issues, including salivary contamination and improper adhesion of the sealant to teeth [3].

To enhance the efficacy of sealants, certain efforts have been directed toward the manipulation of the tooth surface before sealant application [4]. These endeavors encompass both invasive techniques, such as fissurotomy, and non-invasive techniques. The invasive approach involves the use of a drill to enlarge a fissure or air abrasion. This invasive technique allows for superior penetration and adaptation of the sealant compared with conventional untreated fissures [4, 5]. The fissurotomy procedure, along with pumice prophylaxis, has led to controversial results regarding its efficacy in preventing microleakage after PFS [5, 6, 7].

Caries prevention requires a high sealant retention rate, and bonding agents are recommended as noninvasive techniques for this purpose [8]. The integration of a bonding agent layer between a resin sealant and saliva-contaminated enamel may improve the success of all sealant treatments, according to recent in vitro research and a brief clinical investigation [9]. In contrast, another study reported that the retention rate did not increase by using a bonding agent before applying a PFS [10].

Microleakage, as denoted by its definition, pertains to the seepage of microorganisms and fluids through voids that exist between the teeth and the sealant. Microleakage is the leading cause of sealant malfunction [11]. Accordingly, enhancing the success rate of PFS treatment requires the attainment of superior marginal adaptation and more profound occlusal penetration. Owing to the lack of sufficient literature to compare different procedures to reduce microleakage with the use of PFS, the present study was conducted to determine whether bonding agents are more or less effective with regard to microleakage than fissurotomy procedures prior to applying PFSs.

## Materials and methods

Study design

An in vitro study was conducted on 48 freshly extracted, intact first premolars, which were removed for orthodontic reasons. Ethical clearance for the study was obtained from the institutional ethics committee (KDCRC/IEC/2023/56). Teeth with intact occlusal surfaces, free from caries, restorations, and cracks, were included. Teeth with developmental defects were excluded from the study. All teeth were cleaned of debris and blood stains and stored in distilled water at room temperature prior to the testing procedure. The teeth were randomly divided into four groups using a table of random numbers.

Sample size calculation

The sample size was calculated using G*Power v.3.1.9 software. A total of 48 teeth were estimated to achieve a power of 90%, an alpha (α) of 5%, a beta (β) of 10%, a 95% confidence interval (CI), and an effect size of 0.6, as calculated from a previous study [12].

Methodology

**Figure 1:**
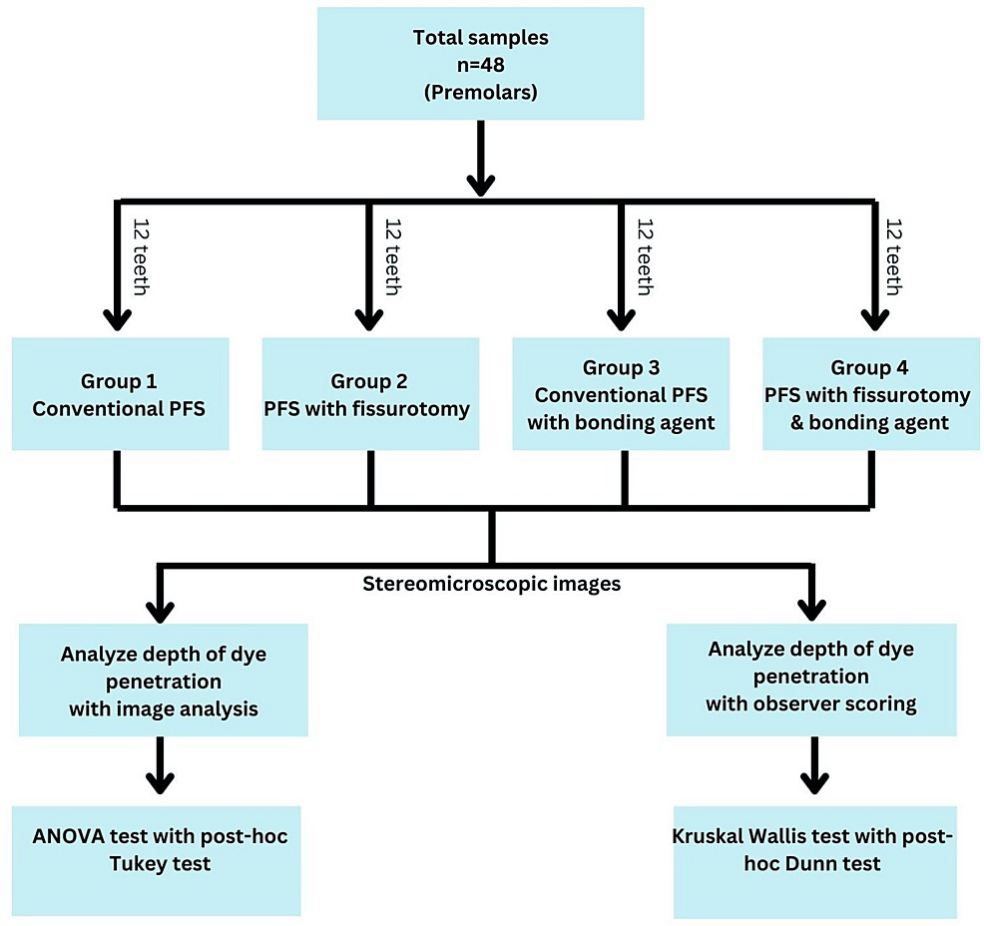
Study design.

Forty-eight teeth were randomly divided into four groups, with 12 teeth in each group, as follows: Group 1 received a conventional PFS (Clinpro, 3M ESPE sealant); Group 2 underwent fissurotomy before PFS placement; Group 3 was treated with a dentin bonding agent (DBA) (Scotchbond Universal Adhesive, 3M ESPE DBA); and Group 4 received both fissurotomy and DBA application before PFS placement (Figure 1).

In Group 1, the occlusal surfaces of all teeth were thoroughly cleaned before PFS placement with a fluoride-free slurry of pumice, using a bristle brush to eliminate accumulated food debris and plaque attached to the teeth. Subsequently, the isolation process was conducted using cotton rolls and adequate suctioning with a four-handed technique to avoid moisture contamination during the procedure. All applications were performed by a single clinician who had undergone training and calibration exercises. The occlusal surfaces of the premolars were treated with 37 percent phosphoric acid etching gel (Scotchbond Universal Etchant, St. Paul, Minnesota, USA) in accordance with the manufacturer's instructions. This gel was applied to the occlusal surfaces for a duration of 20 seconds using a microbrush, followed by a 30-second rinsing period and subsequent drying using a 3-1 syringe for 15 seconds. A light-cure PFS (3M Clinpro Sealant ESPE, St. Paul, Minnesota, USA) was administered, and the sealant was carefully placed on the occlusal surface and meticulously adjusted into the fissures with the aid of a periodontal probe to ensure the absence of any voids or air entrapment during the procedure. Subsequently, the sealant was cured using a light-emitting diode curing light (3M Paradigm DeepCure LED Dental Curing Light) with an output intensity of 1,470 mW/cm² and a wavelength of 430-480 nm. The curing tip was positioned close to the sealant for 20 seconds. The resin-based sealants were scrutinized for voids using a sharp dental explorer, while occlusion was assessed using articulation paper, followed by necessary adjustments using a finishing burnisher.

In Group 2, enamel surface alteration or deepening/widening of fissures of premolars was performed using a fine bur (Diamond CD 57F, Mani, Japan), followed by PFS placement as in Group 1.

In Group 3, after etching with 37% phosphoric acid for 20 seconds, the etchant was washed off, followed by gentle air-drying, and the surface was left slightly moist. Using a micro-applicator, according to the manufacturer's instructions, two coats of DBA were rubbed for 20 seconds, dried using a 3-1 syringe for five seconds, and lightly cured for 10 seconds. Following the solidification process, the primer and adhesive created resin macrotags by infiltrating the area surrounding the enamel prisms. This was followed by PFS placement.

In Group 4, following fissurotomy, DBA was applied prior to PFS placement.

Thermocycling and dye penetration method

All teeth were stored in distilled water for 24 hours prior to testing. They were then exposed to a thermocycling procedure for 15 seconds at each temperature, with a dwell time of 10 seconds between two baths. The temperature ranged between 5°C and 55°C for 1000 cycles to simulate the oral environment. The apices of each tooth sample were subsequently coated with an autopolymerizing acrylic resin, followed by the application of two nail polish coatings on the external layer of each tooth, ensuring a 2 mm clearance around the boundaries of the sealant. The specimens that had undergone coating were immersed in a 1% solution of methylene blue for 24 hours, allowing for the diffusion of the dye into potential crevices between the restoration and the tooth. Following dye exposure, the specimens were subjected to rinsing and washing procedures using distilled water to eliminate any excess coloring material. They were then separated into buccolingual sections as a part of the sealing process, using a high-speed straight handpiece, a diamond disk, and a water mist.

Assessment of microleakage

**Figure 2:**
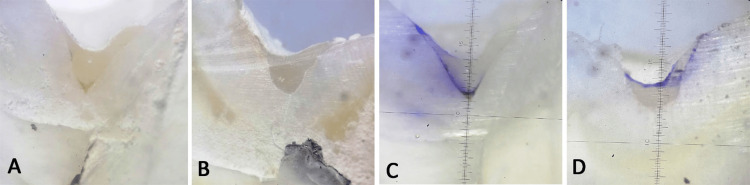
A: Stereomicroscopic images at 40x magnification; B: Before dye penetration; C, D: After dye penetration.

The segmented teeth were assessed under a stereomicroscope (Labomed USA) at a magnification of 40X (Figure 2).

The segmented teeth were assessed under a stereomicroscope (Labomed USA) at a magnification of 40X (Figure 2). Scores were assigned to each sample for microleakage by trained examiners who were blinded to group allocation. All sections underwent an assessment process using the four-point scoring system proposed by Ovrebö RC and Raadal M [13]. The scale for dye penetration was as follows: (0) absence of dye penetration, (1) limited dye penetration to the outer portion of the enamel-sealant interface, (2) dye penetration into the inner portion of the enamel-sealant interface, and (3) dye penetration extending into the underlying fissure. Subsequently, all samples were evaluated for dye penetration in millimeters using image analysis software (Image J, Version 2.3).

Assessment of reliability

Two experienced and trained observers assessed the samples to determine the extent of microleakage, and the average was recorded. After a one-month interval, the observers randomly re-examined 10% of the samples. The reliability values for evaluating the microleakage scores, both within and between observers, were determined using the intraclass correlation coefficient (ICC), yielding results of 0.95 and 0.89, respectively. The accuracy of the observers was also evaluated by comparing their microleakage scores with the assessment of the depth of dye penetration in millimeters, using Pearson’s correlation coefficient, resulting in correlations of 95% for observer 1 and 89% for observer 2 (Table 1).

**Table 1:** Correlation between observer’s estimation of microleakage scores and depth of dye penetration. *P-value less than 0.05 is considered significant.

Observer	r value	P-value
Observer 1	0.953	<0.001>
Observer 2	0.892	<0.001>

Statistical analysis

The data pertaining to microleakage were statistically analyzed for normality using the Shapiro-Wilk test. As the data were found to be normally distributed, the Kruskal-Wallis test was employed, followed by post hoc analysis using the Dunn test to evaluate microleakage scores between the groups. For comparison of dye penetration between the groups, an ANOVA followed by post hoc analysis using Tukey’s test was used. These analyses were conducted using statistical software (SPSS Version 22, Chicago, IL, USA) to identify any significant variations in microleakage. A result was considered statistically significant when a 95% CI was reached.

## Results

The descriptive statistics for different variables, such as dye penetration scores and depth of dye penetration in millimeters, revealed that Group 4 had the lowest scores for both dye penetration and depth of dye penetration, followed by Groups 2, 3, and 1 (Table 2).

**Table 2:** Mean and SD for dye penetration scores and depth. mm: Millimeter.

Variables	Groups	N	Mean
Dye penetration score	1	12	2.16±0.71
	2	12	0.91±0.79
	3	12	1.25±0.75
	4	12	0.50±0.52
Average depth of dye (mm)	1	12	1.18±0.43
	2	12	0.33±0.33
	3	12	0.57±0.40
	4	12	0.11±0.12

A quantitative analysis using the ANOVA test demonstrated statistically significant differences between the groups in terms of dye penetration (p < 0.001), as illustrated in Table 3.

**Table 3:** ANOVA test for comparing depth of dye penetration. *p-value < 0.05: Significant; df: Degrees of freedom.

Cases	Sum of squares	df	Mean square	F value	P-value
Group	7.631	3	2.544	21.25	0.001*
Residuals	5.266	44	0.12		

When utilizing the post-hoc Tukey test for pairwise comparisons, statistically significant differences were observed between Group 1 and all other groups (p = 0.001). Conversely, no significant differences were observed when comparing Groups 2 with 3, and 4 (p > 0.05). Table 4 also revealed statistically significant differences between Groups 3 and 4. This indicated that no statistically significant differences were found in the depth of dye penetration between the use of fissurotomy without DBA and fissurotomy with DBA. However, both procedures significantly reduced microleakage compared to the conventional technique of PFS placement.

**Table 4:** Pairwise comparison of depth of dye penetration by post-hoc analysis using the Tukey test. *p-value < 0.05: Significant; SE: Standard error; NS: Non-significant.

Pairwise comparison of groups	Mean difference	SE	t value	P-value
Groups 1 and 2	0.850	0.141	6.018	0.001*
Groups 1 and 3	0.605	0.141	4.283	0.001*
Groups 1 and 4	1.066	0.141	7.546	0.001*
Groups 2 and 3	-0.245	0.141	-1.735	0.318 (NS)
Group 2 and 4	0.216	0.141	1.528	0.430 (NS)
Group 3 and 4	0.461	0.141	3.263	0.011*

Qualitative analysis showed statistically significant differences in microleakage scores between the groups by the Kruskal-Wallis test. Pairwise comparisons revealed statistically significant differences between groups 1 and 2, groups 1 and 4, and groups 3 and 4. No significant differences were observed between groups 1 and 3, 2 and 3, and 2 and 4 (Tables 5-6). According to the rank, Group 1 showed the maximum reduction in microleakage, followed by Groups 2 and 3. The conventional procedure of PFS placement without prior procedures of fissurotomy or the use of DBA showed the maximum reduction in microleakage.

**Table 5:** Kruskal-Wallis test for comparing scores of depth of dye penetration. *p-value < 0.05: Significant; df: Degrees of freedom.

Groups	Statistic	df	Rank sum	P-value
Group 1	27.919	3	454	0.001*
Group 2			246	
Group 3			308	
Group 4			168	

**Table 6:** Pairwise comparison of depth of dye penetration scores by post-hoc analysis using the Dunn test. *p-value < 0.05: Significant; NS: Non-significant.

Pairwise comparison of groups	Z	W_i_	W_j_	P-value
Groups 1 and 2	3.712	40.083	19.042	0.001*
Groups 1 and 3	2.249	40.083	27.333	0.147 (NS)
Groups 1 and 4	5.035	40.083	11.542	0.001*
Groups 2 and 3	-1.463	19.042	27.333	0.861 (NS)
Groups 2 and 4	1.323	19.042	11.542	1 (NS)
Groups 3 and 4	2.786	27.333	11.542	0.032*

## Discussion

The effectiveness of PFSs depends on their enduring adhesion to the tooth surface over an extended period. A fundamental requirement for this adhesion is the alteration of the enamel surface through the use of an acid conditioning agent or an alternative technique, such as the application of a bonding agent and fissurotomy [1]. The integrity of the interface between the tooth and the sealant is influenced by a multitude of factors, including the anatomy of the fissures, conditions within the oral environment, chemical and mechanical characteristics of the materials employed, and the exertion of masticatory forces [5]. The present study aimed to assess the efficiency of different techniques, such as the use of DBA and fissurotomy, in preventing microleakage with the use of PFSs. The microleakage test, a technique employed to assess the marginal and internal adaptation of restorative materials, utilizes dye penetration as the most commonly utilized and widely acknowledged method for this objective. This method serves as a benchmark in microleakage research and was consequently employed in this study. Stereomicroscopic investigation and image analysis were used to determine the extent of dye penetration. Both qualitative and quantitative methods were used for assessment. For artificial aging, the specimens were subjected to one year of thermocycling.

The present study revealed that PFS, without the prior use of DBA and fissurotomy, showed the maximum microleakage compared to the other groups. Resin-based PFS alters the anatomy of the occlusal surface by sealing retentive pits and fissures, thereby preventing food accumulation, which cannot penetrate pits and fissures for various reasons, such as the salivary pellicle and end products of carbohydrate metabolism. In the present study, thorough cleaning of the occlusal surfaces was performed using pumice prophylaxis [14]. Our study revealed significant differences in microleakage, with and without the use of DBA, aligning with the results of previous studies [1, 9, 15] and contrasting with others [10]. The concept of utilizing a bonding agent beneath the sealant was introduced by Feigal RJ et al. (1993), who employed hydrophilic bonding substances to enhance the adhesive capability when applying the sealant in a moist setting [16]. The contrasting results might be due to the different generations of DBA used in the studies compared to the fourth-generation DBA used in our study. Additionally, unintended excessive dehydration of the etched dentin following acid rinsing significantly increases the probability of collagen mesh collapse, thereby impeding the diffusion of resin monomers within the intertubular dentin. Our study employed a fourth-generation DBA that uses a wet-bonding technique.

With the advancement of fourth-generation DBAs, the concept of hybrid layer formation has been introduced as a mechanism of adhesion to dentin. Nakabayashi first postulated this concept in 1982. This layer is generated by the interpenetration of low-viscosity monomers into the exposed collagen network and intertubular dentin, establishing a micromechanical bond with the dentin. When appropriately established, the hybrid layer exhibits exceptional strength and resilience, providing substantial micromechanical retention for resin composites [17].

The present study revealed that the fissurotomy procedure resulted in less microleakage compared to the conventional PFS placement technique, aligning with findings from previous studies [4, 6, 7, 18]. It is postulated that the enhanced preservation can be attributed to the augmentation of depressions and crevices, resulting in greater surface area for adhesion and the application of a denser sealant coating, which likely exhibits increased durability. Additionally, fissurotomy enhances the permeation of the sealant due to the broadening and deepening of depressions and crevices, and the removal of organic substances and plaque [7]. The benefits of fissurotomy on microleakage may also be attributed to its impact on prismless enamel. Gwinnett AJ found that the presence of a prismless layer on the enamel surface could potentially decrease the mechanical adhesion of the sealant. In areas where the prismless enamel was present, no resin tags indicating sealant penetration were observed; however, they were present in areas where the enamel had been removed. When present, the length of resin projections associated with prismless enamel was notably shorter than those associated with prismatic enamel, as observed by scanning electron microscopy [19].

The group in which fissurotomy was performed in conjunction with DBA prior to PFS application demonstrated the lowest level of microleakage. Nevertheless, the use of DBA, with or without fissurotomy, did not result in a significant reduction in microleakage. The findings of this in vitro comparative investigation suggest that fissure preparation should be performed using a bur, followed by phosphoric acid etching, aligning with previous studies [20, 21]. This procedure potentially enhances the durability of the sealant as it improves the depth of penetration and reduces marginal leakage. According to a previous study, the use of a bonding agent as a mediator between the enamel and sealant did not influence the success of the sealant unless there were instances where satisfactory isolation and contamination control were unachievable [22]. Given that this investigation was conducted in a controlled laboratory setting and excluded the presence of saliva contamination, it is plausible that the application of any bonding agent would not have a statistically significant impact on the occurrence of sealant microleakage. In this study, we used a low-viscosity sealant (Clinpro, 3M ESPE). Previous studies have observed that a low-viscosity sealant has the ability to completely infiltrate the etched surface, resulting in the formation of a resin-containing layer that extends beyond the depth of the etching in the enamel [21]. Some differences were noted in the results obtained by observers for microleakage scores and assessment of dye penetration depth using image analysis software, which could be attributed to differences in observer accuracy, as observer one demonstrated greater accuracy than observer two.

Limitations of the study

One limitation of this study was the absence of a cutting machine with finer blades, which would have allowed for more slices per tooth and a more thorough assessment of microleakage. Additionally, as with other in vitro studies, it is important to supplement these findings with clinical studies. Well-designed randomized controlled trials should be conducted to test the results of the present study on patients, with long-term follow-up of up to two years.

## Conclusions

Fissurotomy significantly reduced microleakage, with or without the prior use of a DBA, before the placement of PFSs. The use of a DBA alone also significantly reduced microleakage compared to the conventional procedure for placing PFSs without DBA. In situations where controlling saliva flow and achieving isolation are not feasible, using a bonding agent can be advantageous for enhancing the efficacy of fissure sealant therapy.
